# Clinical and Epidemiologic Characterization of WU Polyomavirus Infection, St. Louis, Missouri

**DOI:** 10.3201/eid1312.070977

**Published:** 2007-12

**Authors:** Binh-Minh Le, Lee M. Demertzis, Guang Wu, Robert J. Tibbets, Richard Buller, Max Q. Arens, Anne M. Gaynor, Gregory A. Storch, David Wang

**Affiliations:** * Washington University School of Medicine, St. Louis, Missouri, USA

**Keywords:** WU polyomavirus, Polyomaviridae, respiratory tract infection, viral persistence, dispatch

## Abstract

WU polyomavirus is a recently described polyomavirus found in patients with respiratory infections. Of 2,637 respiratory samples tested in St. Louis, Missouri, 2.7% were positive for WU polyomavirus by PCR, and 71% were coinfected with other respiratory viruses. Persistent human infection with WU polyomavirus is described.

An initial report described the identification of WU polyomavirus in 6 (0.7%) of 890 respiratory tract samples collected in St. Louis, Missouri, USA, and in 37 (3.0%) of 1,245 respiratory tract specimens tested from Brisbane, Queensland, Australia (*1*). The goal of our study was to extend these initial findings by determining the prevalence of WU polyomavirus in a larger patient cohort in St. Louis.

## The Study

We tested 2,637 nasopharyngeal swabs or nasal washes (from patients 1 day to 88 years of age) submitted to the virology laboratory at St. Louis Children’s Hospital for routine respiratory virus detection from July 2003 through June 2004. Of these samples, 2,263 were from children <4 years of age (including 419 newborns) and 374 were from children >4 years of age. The specimens were extracted with the automated Roche MagNA Pure LC extractor and MagNA Pure LC Total Nucleic Acid Isolation Kit (Roche Diagnostics, Indianapolis, IN, USA).

For real-time PCR, amplification primers WU-TAB02-F 5′- tgttgcatccatttgttacattcat-3′ and WU-TAB03-R 5′-GAAAGAACTGTTAGACAAATATATAGGCCTTA-3′ and the minor groove binder probe WU-TAB04-pro 5′-6FAMatgtcagcaaattcMGBNFQ-3′ were used with a commercially available universal TaqMan real-time PCR master mix and ABI 7500 Real-Time Thermocycler (Applied Biosystems, Foster City, CA, USA). All WU polyomavirus–positive specimens were screened for 17 additional viruses (influenza A and B; RSV A and B; PIV 1–4; human metapneumovirus; adenovirus subgroups B, C, and E; rhinovirus; and coronaviruses OC43, 229E, and NL63) by using the EraGen MultiCode-PLx respiratory virus panel as described previously (*1*).

Each clinical specimen was assigned a code. Collection of clinical data was approved by the Washington University Human Research Protection Office. Pertinent demographic, historical, and clinical information, when available, was collected by using a standard collection form. Statistical significance was determined by using 2-tailed Fisher exact χ^2^ tests with Epi Info software version 3.4 (Centers for Disease Control and Prevention, Atlanta, GA, USA )

Seventy (2.7%) of the 2,637 tested specimens were positive for WU polyomavirus; 71% of the positive samples were also positive for >1 other respiratory virus. Of the 70 positive samples, 5 were omitted from analysis because of chart unavailability. The remaining 65 samples were collected from 60 individual patients (5 specimens were serial samples associated with distinct clinical syndromes in 2 immunocompromised patients).

Of the 60 WU-positive patients, 31 (52%) were female. The ethnic breakdown was as follows: 50% African-American, 47% Caucasian, 3% other. Positive specimens were noted for patients 1 day to 15 years of age (Appendix Table). The highest and lowest rates of infection are displayed in the Figure, panel A.

**Figure:**
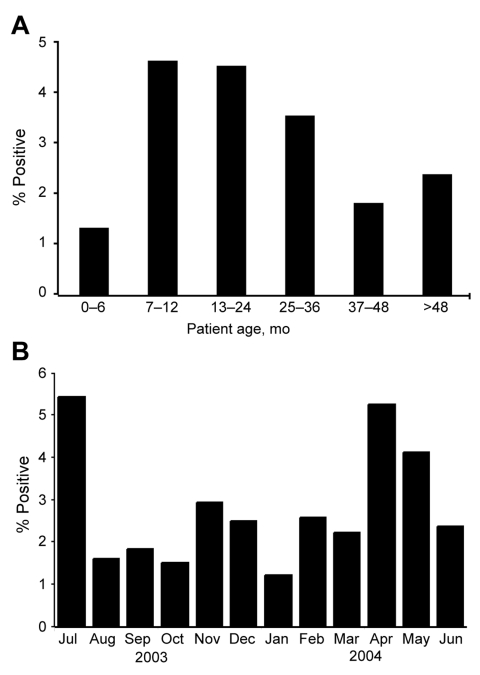
A) Percentage of samples positive for WU polyomavirus by age group. B) Percentage of samples positive for WU polyomavirus by month.

Patients positive for WU polyomavirus were detected throughout the year. A small peak was observed in July 2003, and a second small peak was observed in April and May 2004 (Figure, panel B). WU polyomavirus was the only virus detected in a 1-day-old full-term infant delivered by cesarean section who had been transferred to St. Louis Children’s Hospital with respiratory distress requiring intubation. He was afebrile with lung opacities on chest radiograph. Patent ductus arteriosus and pulmonary hypertension were eventually diagnosed.

The 3 oldest patients positive for WU polyomavirus in this cohort were immunosuppressed. They included a 12-year-old with Evans syndrome and a 15-year-old with severe combined immunodeficiency syndrome (both post–bone marrow transplant) and a 14-year-old with end-stage renal disease and asthma.

The most common clinical findings in the patients with WU polyomavirus are listed in the Table. The most frequent diagnoses were pneumonia (31%) (although 40% had positive bacterial cultures), bronchiolitis (25%), and upper respiratory tract infections (15%). We also compared all the measured parameters from the patients who were infected with WU alone to the patients who were co-infected with other viruses. In most cases, no statistically significant differences occurred between the co-infected and WU polyomavirus–only patients, except that more co-infected patients than WU polyomavirus–only patients had rhinorrhea (23/47 vs. 2/18; p = 0.005) and upper respiratory tract symptoms (30/47 vs. 6/18; p = 0.049). In addition, significantly more children with co-infection than with only WU polyomavirus had prior daycare exposure (18/45 vs. 1/15; p = 0.02).

**Table:** Clinical parameters in episodes of WU polyomavirus infection

Symptoms and physical examination findings (n = 65)	%
Symptoms	
Cough	57
Upper respiratory tract symptoms	55
Rhonchi/crackles/coarse breath sounds	46
Shortness of breath or increased work of breathing	42
Wheezing	40
Rhinorrhea	38
Retractions	37
Decreased oral intake	34
Vomiting	32
Diarrhea	18
Stridor	6
Rash	6
Apnea	5
Signs	
Tachypnea* (n = 57)	79
Hypoxia† (n = 55)	47
Fever‡ (n = 63)	41
Bandemia (n = 42)	40
Leukocytosis§ (n = 42)	31
Leukopenia¶ (n = 42)	10
Radiographic or computed tomographic findings (n = 50)	
Infiltrate or consolidation	72
Hyperinflation	14
Peribronchial cuffing	12
Effusions	6
Treatment	
Antimicrobial agents (n = 65)	58
Bronchodilators (n = 65)	38
Steroids (n = 65)	37
Oxygen (n = 65)	25
Intubation (n = 63)	11
Intensive care (n = 63)	19
Risk factors	
Daycare	32
Sick contacts	25
Medical history	
Asthma (n = 60)	37

*Defined per National Institutes of Health clinical center guidelines. †SaO_2_ <95% or PaO_2_ >80%. ‡Temperature >38°C. §Leukocyte count >15,000/mm^3^. ¶Leukocyte count <4,000/mm^3^.

The cohort of 2,637 samples included several sets of sequential samples taken from the same patient during the course of prolonged illness. In 2 patients, sequential samples obtained over a span of 6–8 weeks were positive for WU polyomavirus. The first patient was a 4-year-old girl with hemophagocytic lymphohistiocytosis, who had 4 distinct respiratory specimens that tested positive during a 2-month period. Her first positive specimen was obtained while she was asymptomatic during admission for a bone marrow transplant in September 2003. A second sample was obtained during a clinic visit for nasal congestion and cough in November 2003. Her third sample (also positive for coronavirus OC43) was obtained during an admission 5 days later for pneumonia. Finally, a fourth sample (negative for the previously detected coronavirus OC43) was taken 7 days later during the same admission after an episode of apnea.

The second patient was a 16-month-old child with biliary atresia admitted for a liver transplant in September 2003. He had 3 positive samples during a 6-week period. An initial sample taken on admission was negative for WU polyomavirus. Six weeks after transplant, fever and shortness of breath requiring intubation developed. A sample taken then was positive for both WU polyomavirus and adenovirus. Four weeks later, worsening shortness of breath and fever developed. His blood cultures were now also positive for *Klebsiella* and *Enterobacter* spp., and his third respiratory sample demonstrated both WU polyomavirus and rhinovirus. Two weeks later, fever, hypoxia, and increasing secretions developed, with his fourth sample positive only for WU polyomavirus. Followup samples obtained 3 months later for each patient indicated clearance of WU virus.

## Conclusions

Patients infected with WU polyomavirus in this cohort were primarily hospitalized with pneumonia, bronchiolitis, and upper respiratory tract infections. One new observation is that multiple respiratory specimens sampled from the same patient over 6–8 weeks were positive for WU polyomavirus, which suggests that WU polyomavirus may persistently infect humans. Both patients were immunocompromised, although they were able to clear infections with other viruses (coronavirus OC43, rhinovirus, adenovirus), which suggests that the continued detection of WU polyomavirus was not due to a completely incapacitated immune system. Sequence analysis of a 250-bp fragment of the VP2 gene of WU polyomavirus amplified from multiple samples from the 2 patients showed no sequence polymorphisms between the initial and later samples (data not shown). As some sequence variation has previously been reported in this locus (*1*), these data are consistent with the model of persistent infection. The detection of WU polyomavirus in the respiratory secretions of a 1-day-old infant suggests that vertical transmission of WU polyomavirus from mother to fetus may occur, although further studies are needed to verify this suggestion.

In conclusion, WU polyomavirus was detected in 2.7% of patients with respiratory tract infections. A high percentage of coinfection with other respiratory viruses was detected, complicating interpretation of the clinical findings. However, WU polyomavirus was the sole virus detected in 20 specimens from patients with respiratory illness, which suggests that it may be a respiratory pathogen. Finally, the observed persistence of this virus suggests analogy to BK and JC viruses in this regard (*2*).

## Supplementary Material

Appendix Table Specimens positive for WU polyomavirus, July 2003 through June 2004*
