# Supplementary material for: Clinical and Epidemiologic Characterization of WU Polyomavirus Infection, St. Louis, Missouri
Source: Emerg Infect Dis. 2007 Dec;13(12):1936–8. doi: 10.3201/eid1312.070977 (PMC2876771; doi:10.3201/eid1312.070977)
Supplement: Appendix Table — Specimens positive for WU polyomavirus, July 2003 through June 2004* [file 07-0977_appT-s1.pdf]

Appendix Table. Specimens positive for WU polyomavirus, July 2003 through June 2004*

Specimen ID	Month	Patient age, y†	Co-infection	Clinical manifestations‡	Duration of hospitalization
S7	Jul	12	Rhino	Pneumonia	23 d
S8	Jul	0.8	PIV4	Bronchiolitis	<24 h
S9	Jul	0.3	None	Pneumonia	9 d
S10	Jul	0.9	PIV1, PIV3, Adeno C, Rhino	Bronchiolitis	3 d
S11	Jul	2	None	Bronchiolitis/pneumonia	7 d
S12	Jul	2	None	Pneumonia	9 d
S13	Jul	15	Rhino	Pneumonia	2 d
S14	Jul	14	None	Pneumonia	1 d
S15	Aug	1	RSVa	Bronchiolitis	1 d
S16	Aug	2	RSVa	Bronchiolitis/pneumonia	10 d
S17	Sep	1	None	Croup	2 d
S18	Sep	4	None	Pretransplant admission	23 d
S19	Sep	1	PIV1, Rhino	Croup/pneumonia	2 d
S20	Oct	0.8	RSVa	Bronchiolitis/pneumonia	<24 h
S21	Oct	2	Rhino	Bronchiolitis/pneumonia	4 d
S22	Oct	0.8	Adeno C, OC43	Upper respiratory tract infection, strep pharyngitis	3 d
S23	Oct	0.5	RSV	No clinical data available	NA
S24	Nov	0.6	RSVa	No clinical data available	NA
S25	Nov	4	None	Upper respiratory tract infection	Outpatient clinic visit
S26	Nov	1	AdenoC	Pneumonia	106 d
S27	Nov	4	OC43	Pneumonia	28 d
S28	Nov	0.8	HBoV	Pneumonia	<24 h
S29	Nov	4	None	Gastroenteritis	28 d
S30	Nov	2	InflA, RSVa	Upper respiratory tract infection	<24 h
S31	Nov	1	None	Pneumonia	8 d
S32	Dec	1 d	None	Pneumonia	26 d
S33	Dec	0.5	None	Upper respiratory tract infection, cellulitis with abscess	1 d
S34	Dec	1	Rhino	Pneumonia	106 d
S35	Dec	1	None	Pneumonia	106 d
S36	Dec	3	InflA, HBoV	Upper respiratory tract infection, Influenza	1 d
S37	Dec	1	OC43	Bronchiolitis/pneumonia	2 d
S38	Dec	0.3	InflA	Bronchiolitis/pneumonia	3 d
S39	Jan	0.5	None	No clinical data available	NA
S40	Jan	1	RSVa	Pneumonia	Outpatient clinic visit
S41	Feb	1	RSVa	Bronchiolitis/pneumonia	2 d
S42	Feb	1	None	No clinical data available	NA
S43	Feb	1	hMPVb	Upper respiratory tract infection	3 d
S44	Feb	2	RSVa, Rhino	Bronchiolitis/pneumonia	1 d
S45	Feb	0.1	None	Tracheoesophageal fistula, hyaline membrane disease	50 d
S46	Feb	2	Adeno C	Tracheobronchitis, enterocolitis	4 d
S47	Feb	0.7	hMPV	No clinical data available	NA
S48	Mar	1	None	Bronchiolitis	1 d
S49	Mar	1	None	Bronchiolitis	2 d
S50	Mar	2	Rhino	Upper respiratory tract infection	2 d
S51	Mar	3	PIV3, Rhino	Bronchiolitis	2 d
S52	Mar	0.8	hMPV	Bronchiolitis	2 d
S53	Mar	0.8	hMPV	Bronchiolitis	2 d
S54	Apr	0.8	hMPV	Pneumonia	1 d
S55	Apr	2	Adeno E	Pneumonia	5 d
S56	Apr	2	hMPVa	Febrile seizures	2 d
S57	Apr	1	Adeno C	Croup	1 d
S58	Apr	0.4	Rhino, PIV4b	Bronchiolitis	<24 h
S59	Apr	1	None	Diarrhea with volume depletion	7 d
S60	Apr	0.8	hMPVb, HBoV	Pneumonia	<24 h

S61	Apr	3 wks	Rhino	Tracheobronchitis	1 d
S62	Apr	0.3	hMPV	Bronchiolitis	2 d
S63	Apr	1	hMPV	Bronchiolitis	1 d
S64	Apr	0.3	Rhino	Bronchiolitis	1 d
S65	Apr	4	Adeno A	Upper respiratory tract infection	3 d
S66	May	0.5	Adeno	Pneumonia	2 d
S67	May	0.3	None	Bronchiolitis	1 d
S68	May	0.3	Rhino	Bronchiolitis	1 d
S69	May	0.8	PIV3, Rhino	Bronchiolitis/pneumonia	2 d
S70	May	4	None	Upper respiratory tract infection	2 d
S71	May	0.4	PIV, Rhino	Pneumonia	1 d
S72	May	2	Rhino, CMV	Bronchiolitis	1 d
S73	Jun	3	PIV3	Pneumonia	12 d
S74	Jun	2	Rhino	Upper respiratory tract infection	1 d
S75	Jun	0.8	Rhino	Pneumonia	3 d
S76	Jun	2	Rhino	Bronchiolitis	2 d

*Rhino, rhinovirus; PIV, parainfluenza virus; Adeno, adenovirus; RSV, respiratory syncytial virus; NA, not available; Strep, *Streptococcus*; HBoV, human bocavirus; InflA, influenza A; hMPV, human metapneumovirus; CMV, cytomegalovirus.
†Unless otherwise stated.
‡Clinical diagnoses determined according to the criteria of Denny and Clyde, when applicable (*1*).

Reference

1. Denny FW, Clyde WA Jr. Acute lower respiratory tract infections in nonhospitalized children. J Pediatr. 1986;108:635–46.
